# Construction and Validation of a 90-Day Mortality Risk Prediction Model Based on Interpretable Machine Learning for Acute Ischemic Stroke Patients Undergoing Mechanical Thrombectomy

**DOI:** 10.3390/jcm15124702

**Published:** 2026-06-17

**Authors:** Qian Jiang, Rui Wang, Yueyue He, Lingxiao He, Nan Wen, Jianyu Peng, Junli Zhang, Ling Feng

**Affiliations:** 1 Department of Neurology, West China Hospital, Sichuan University/West China School of Nursing, Sichuan University, Chengdu 610041, China; 15608088752@163.com (Q.J.); hxwangrui2020@163.com (R.W.); heyueyue0501@163.com (Y.H.); wn20208558@163.com (N.W.); 15196788875@163.com (J.P.); 2 Department of Emergency, West China Tianfu Hospital, Sichuan University, Chengdu 610213, China; 3 Trauma Centre of West China Hospital, Sichuan University, Chengdu 610041, China; helingxiao@wchscu.cn; 4 Department of Neurology, West China Tianfu Hospital, Sichuan University, Chengdu 610213, China; 5 Department of Gastroenterology, West China Hospital, Sichuan University/West China School of Nursing, Sichuan University, Chengdu 610041, China; 19828057809@163.com

**Keywords:** acute ischemic stroke, mechanical thrombectomy, 90-day mortality risk, predictive models, cohort studies, machine learning, Shapley additive explanation algorithm

## Abstract

**Background/Objectives**: The accurate prediction of postprocedural mortality is critical for clinical decision-making; however, research on mortality risk models for patients undergoing mechanical thrombectomy remains limited. This study aimed to develop and validate machine learning models for predicting 90-day post-mechanical thrombectomy mortality. **Methods**: A retrospective-prospective cohort study involving 699 retrospective patients (January 2019–December 2022) and 274 prospective patients (January 2023–June 2024) from a single institution in Sichuan was conducted. The primary outcome was all-cause mortality within 90 days, ascertained via telephone follow-up. Predictors were identified using univariate analysis and LASSO regression. Eight predictive models were developed and evaluated using existing machine learning methods via 10-fold cross-validation. Model performance was assessed through discrimination, calibration, decision curve analysis, and interpretability via Shapley additive explanations. **Results**: The final dataset included 593 patients in the modeling set and 247 in the validation set. The 90-day mortality rates were 25.6% and 32.0%, respectively. Key predictors included age, hyperlipidemia, atrial fibrillation, pre-stroke statin use, antiplatelet/anticoagulant therapy within 48 h of onset, dysphagia, D-dimer levels, and activities of daily living scores. Logistic regression demonstrated superior performance in the modeling cohort (AUC = 0.87), whereas the multilayer perceptron model exhibited the greatest efficacy in the validation cohort (AUC = 0.77). **Conclusions**: Machine learning algorithms can accurately predict 90-day mortality among patients undergoing mechanical thrombectomy. The multilayer perceptron model demonstrated robust validation performance and offers a potential tool for personalized risk assessment and optimization of clinical decision-making.

## 1. Introduction

Stroke is the second leading cause of mortality among non-communicable diseases worldwide, affecting approximately 11.9 million individuals annually and resulting in nearly 5 million deaths or instances of disability [1,2]. In China, stroke is the primary cause of adult mortality and disability, with the nation reporting the highest global incidence rate of 39.9%. More than 2 million new stroke cases and 1.9 million stroke-related fatalities are recorded in China annually [3]. Driven by the aging global population and the increasing incidence of chronic conditions, stroke represents a significant threat to public health.

Acute ischemic stroke (AIS) accounts for 69.6% to 72.8% of new stroke cases in China and poses a considerable public health burden [4]. In recent years, mechanical thrombectomy (MT) has emerged as a cornerstone treatment for AIS, facilitating rapid recanalization by physically extracting the thrombus to restore cerebral perfusion [5]. Meta-analyses of landmark trials, including RESCUE-Japan LIMIT, ANGEL-ASPECT, and SELECT2, have demonstrated that compared with pharmacologic therapy alone, MT significantly improves 90-day survival and functional outcomes, even in patients with large infarct cores [6]. Despite the adoption of MT as the standard of care, post-treatment mortality remains a critical concern, with highly variable clinical prognoses [7]. Reports indicate that post-MT morbidity and mortality rates range from 18.4% to 38.2%, highlighting a persistently high-risk profile [8,9,10,11]. Consequently, accurate prediction of mortality risk in MT patients is essential for optimizing treatment strategies and enhancing clinical outcomes. Given that ML has successfully improved risk stratification in other fields—such as predicting lung cancer risk in individuals undergoing low-dose CT screening [12]—there is a clear opportunity to apply these methods to prognostic assessment following MT.

Machine learning (ML) is an interdisciplinary field that integrates statistics and computer science and uses sophisticated algorithms and large-scale datasets to facilitate classification, prediction, and the discovery of latent patterns. ML has been extensively applied across various sectors, particularly in healthcare [13]. With rapid technological advancements, ML has become an essential tool for predicting stroke risk because of its ability to model complex data relationships and high-dimensional features [14,15]. Numerous studies have developed ML-based models to predict post-stroke mortality [16,17]. However, mortality prediction models specifically developed for patients undergoing MT remain limited. Current predictive models for MT often fail to adequately incorporate procedure-specific variables that may significantly influence prognosis, such as the number of thrombectomy passes, surgical techniques, and anesthesia grading [18]. Furthermore, the majority of contemporary models are derived from single-center datasets with limited sample sizes and lack robust external validation, which restricts their generalizability and clinical applicability [19]. Finally, many existing clinical prediction models lack interpretability, providing insufficient decision support for clinicians and thereby hindering their implementation in real-world settings [20].

This study aimed to develop and temporally validate ML models for predicting 90-day all-cause mortality in patients with AIS treated with MT. By integrating demographic factors, clinical characteristics, medication usage, procedural data, and laboratory indicators, we sought to identify clinically significant predictors of mortality. Furthermore, we employed Shapley Additive Explanations (SHAP) analysis to elucidate the contribution of specific variables to model predictions, thereby enhancing model transparency and facilitating personalized risk stratification in clinical practice.

## 2. Materials and Methods

### 2.1. Research Design and Participants

Data for the model development cohort were retrospectively gathered from patients with MT treated from January 2019 to December 2022. Temporal validation was conducted utilizing a prospective cohort of patients treated between January 2023 and June 2024. This research was performed in the Department of Neurology at a major general hospital in Sichuan, China, which functions as a stroke center in southwestern China and offers representative data on MT patients. This research complied with the TRIPOD+AI reporting standard [21]. The clinical characteristics of the entire cohort and the results of the intergroup comparisons are presented in Table 1.

The inclusion and exclusion criteria were applied consistently across both cohorts. Patients were included if they met the following criteria: (1) age ≥ 18 years; (2) diagnosis of acute ischemic stroke (AIS) in accordance with the 2023 Chinese Guidelines for the Diagnosis and Treatment of AIS, confirmed by cranial CT or MRI; (3) candidacy for MT; (4) provision of informed consent for emergency MT; and (5) completion of laboratory tests within 24 h of admission, with available follow-up data obtained via in-person visits or telephone interviews. Patients were excluded if they met any of the following criteria: (1) the presence of pre-existing severe intracranial dysfunction or genetic disorders; (2) end-stage organ failure (e.g., cardiac, pulmonary, hepatic, or renal) or critical conditions such as malignant neoplasms or severe hematological disorders; (3) concurrent acute extracerebral arterial embolism; (4) pregnancy; (5) a modified Thrombolysis in Cerebral Infarction (mTICI) score < 2b; (6) inability to access complete medical records from the electronic system; or (7) incomplete data hindering effective analysis.

### 2.2. Sample Size

The sample size for the model development cohort was determined using the dichotomous clinical prediction model approach developed by Riley et al., implemented via the pmsampsize package [22]. On the basis of a literature-derived event rate of approximately 38% and the inclusion of 10 candidate predictors, a sample size of 363 was required to achieve an area under the curve (AUC) of at least 0.8. The validation cohort size was determined using the minimum sample size formula for the external validation of dichotomous clinical prediction models [23]. On the basis of an anticipated mortality rate of 0.38, an average AUC of 0.75, an expected calibration slope of 0.8, an observed/expected (O/E) statistic error <0.05, and a clinical probability threshold of 0.3, a minimum of 171 cases was needed.

### 2.3. Research Indicators

Predictors were selected on the basis of an extensive literature review and expert consultation. Clinical data included the following: (1) Demographics: age, gender, ethnicity, body mass index (BMI), education level, and smoking/alcohol history; (2) Disease characteristics: admission vital signs, TOAST classification, vascular occlusion site, stroke severity [National Institutes of Health Stroke Scale (NIHSS): <4 (mild), 4–15 (moderate), 16–24 (severe), and ≥25 (very severe)], admission modified Rankin Scale (mRS) score, Barthel Index (ADL), and presence of dysphagia; (3) Medication: pre-stroke statin, anticoagulant, or antiplatelet use, and initiation of antithrombotic therapy within 48 h of onset; (4) Procedural data: bridging therapy, thrombectomy technique, number of passes, anesthesia method and classification [American Society of Anesthesiologists (ASA) Physical Status], onset-to-puncture time (OPT), puncture-to-reperfusion time (PNT), and adjunct procedures (endotracheal intubation, balloon dilation, or stenting); and (5) Laboratory measures: albumin, lymphocyte, C-reactive protein (CRP), total cholesterol, monocyte, neutrophil, platelet, uric acid, D-dimer, fibrinogen, triglycerides, low-density lipoprotein cholesterol, and high-density lipoprotein cholesterol. The primary outcome was 90-day all-cause mortality.

### 2.4. Data Collection

Data were extracted from the hospital information system by two trained professionals. Clinical nurses conducted outcome follow-up via telephone 90 days post-stroke. A neurologist assessed disease-specific metrics and procedural data, including the TOAST classification, mRS score, and NIHSS score.

### 2.5. Data Preprocessing and Statistical Analysis

Variables with >20% missing values were excluded. Missing data for the remaining variables were imputed using the missForest algorithm (5 iterations) via the R mice package. MissForest is an iterative imputation technique that uses random forest, which forecasts missing values by developing a nonparametric ensemble model from other observable variables and progressively refining the estimates over successive iterations until convergence [24]. The Kolmogorov–Smirnov test was used to evaluate data normality. Normally distributed data are presented as the mean ± standard deviation, whereas nonnormally distributed data are presented as medians with interquartile ranges (25th and 75th percentiles). Categorical variables are presented as frequencies and percentages. A univariate study was conducted to determine characteristics correlated with mortality in patients who underwent MT. Due to the nonnormal distribution of continuous data, the Mann–Whitney U test was employed for group comparisons. Categorical variables were examined utilizing either the χ^2^ test or Fisher’s exact test, as deemed appropriate. LASSO regression analysis was performed utilizing glmnet software (version 4.1-8) to identify pertinent factors and mitigate multicollinearity. All the statistical analyses were conducted with R software (version 4.3.2). A two-tailed *p* value of less than 0.05 was considered to indicate statistical significance.

### 2.6. Modeling

**Figure 1:**
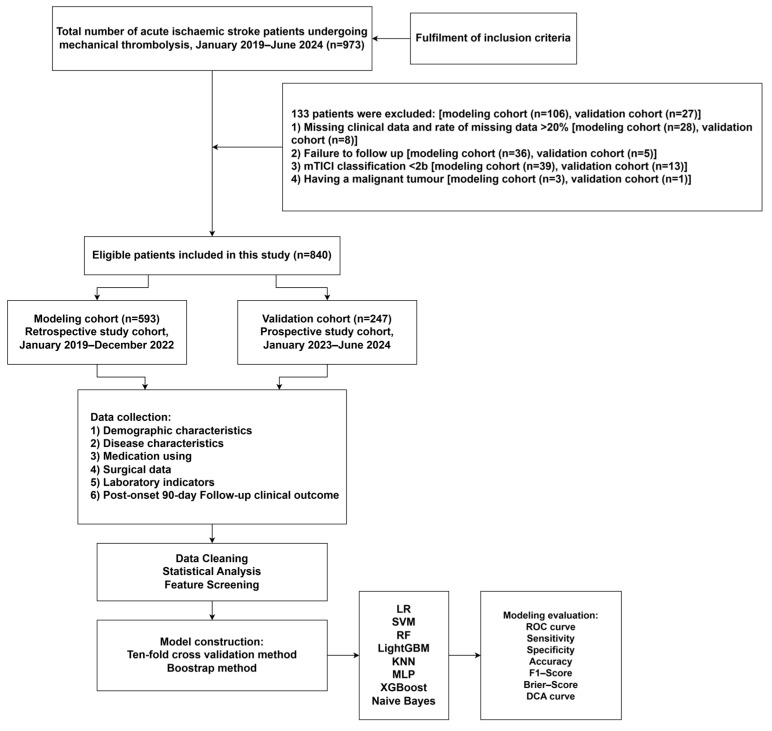
Workflow diagram for this study.

Model construction was performed in Python 3.13. Eight ML algorithms were evaluated: logistic regression (LR), support vector machine (SVM), random forest (RF), light gradient boosting machine (LightGBM), k-nearest neighbors (KNN), multilayer perceptron (MLP), eXtreme Gradient Boosting (XGBoost), and naive Bayes. Hyperparameter optimization was conducted via the Optuna framework. To ensure reproducibility, random seeds were fixed for data partitioning and training. To reduce the risk of overfitting and improve model robustness, 10-fold cross-validation was applied during both model development and validation performance assessment. Model performance was evaluated using the AUC, sensitivity, specificity, accuracy, F1 score, calibration slope/intercept, and Brier score. Discrimination, calibration, and clinical utility were assessed via receiver operating characteristic (ROC), calibration, and decision curve analysis (DCA), respectively. Finally, the model with the best overall performance in the temporal validation cohort was interpreted using SHAP. The patient selection and modeling process are shown in Figure 1.

## 3. Results

### 3.1. Baseline Data

**Figure 2:**
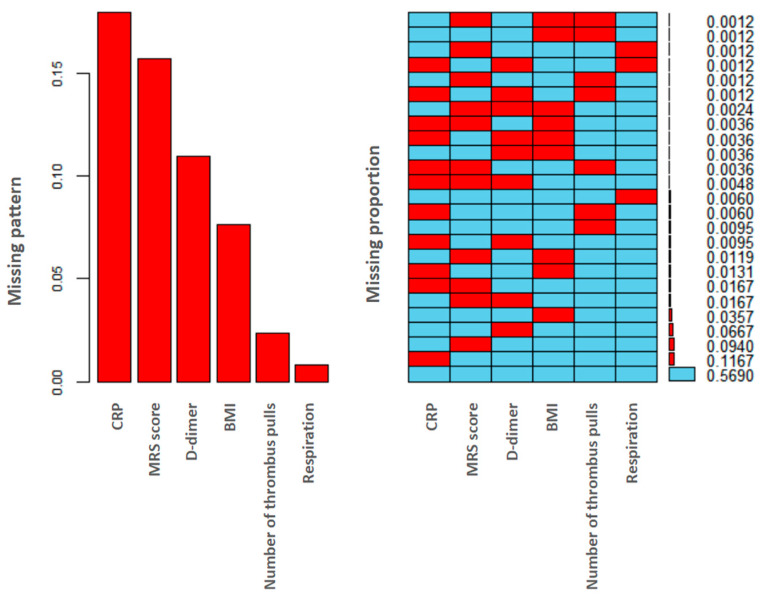
Missing pattern and proportion.

Between January 2019 and December 2022, a total of 699 patients with MT were included in the modeling cohort. Following the exclusion of 106 patients, data from 593 patients were used for modeling purposes. Temporal validation data were gathered from January 2023 to June 2024 and included 274 patients. After 27 individuals were excluded, the validation cohort comprised 247 participants. This investigation involved the imputation of 463 missing variables. Five comprehensive datasets were generated using MissForest multiple imputation, with the missing patterns and variable proportions illustrated in Figure 2. The absence rates for all included variables were under 20%, namely, CRP (17.98%), mRS score (15.71%), D-dimer (10.95%), BMI (7.26%), number of thrombus pulls (2.38%), and respiration (0.83%).

In the modeling cohort, there were 152 patient fatalities (25.6%), while in the temporal validation cohort, 79 fatalities (32%) were documented. The univariate analysis results of mortality in patients with MT in the modeling cohort revealed that numerous factors significantly predicted postoperative death, including age, gender, hyperlipidemia, atrial fibrillation, other cardiac conditions, the NIHSS score, the ADL score, admission dysphagia, prior use of antiplatelet drugs, statins, and anticoagulants, as well as antiplatelet/anticoagulant therapy administered within 48 h, thrombectomy methods, the number of thrombus pulls, anesthesia grading, PNT, balloon dilation/stenting, albumin levels, lymphocyte counts, CRP levels, and D-dimer levels (*p* < 0.05). Table 1 illustrates the distribution of particular features between the surviving and deceased groups. A comparison of baseline data between the modeling cohort and the temporal validation set, as illustrated in Table 2, identified five distinct variables: admission ADL score, albumin, CRP, monocyte count, and D-dimer, but the other characteristics did not demonstrate significant variations.

### 3.2. Screening of Predictive Features

**Figure 3:**
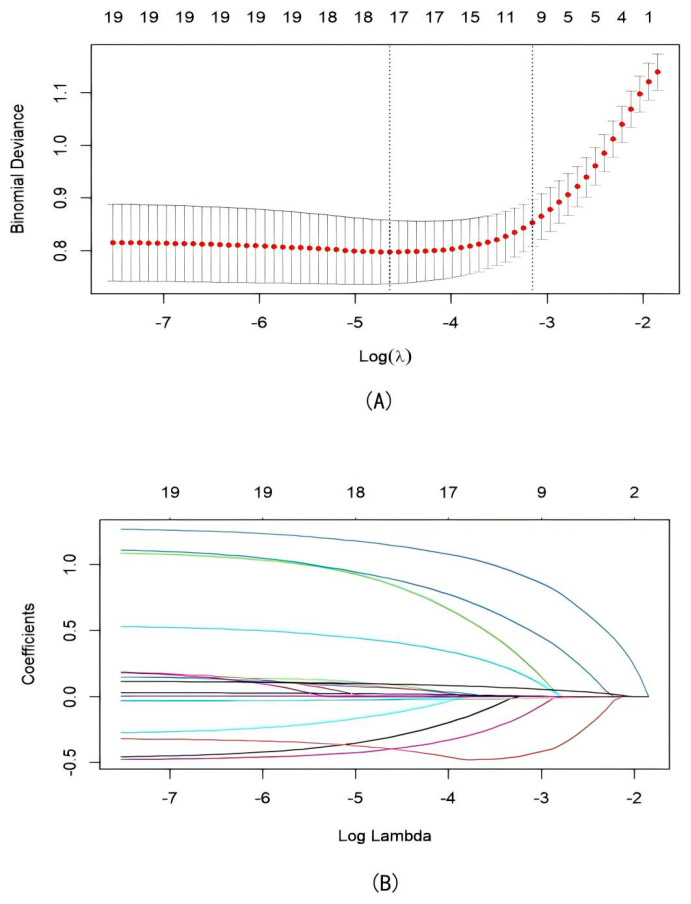
LASSO regression variable screening: (**A**) selection process of parameter λ; (**B**) dynamic process of λ with variables.

LASSO regression analysis was employed to identify the 20 clinically important factors from the univariate study. The binomial deviation obtained from cross-validation plotted against log(λ) is shown in Figure 3A. The numbers above the graphic reflect the number of variables kept in the model under different penalty strengths. The coefficient profiles of the variables are shown in Figure 3B, with the *y*-axis denoting the regression coefficients and the *x*-axis indicating log(λ). As λ increases, the regularization constraint intensifies, causing the coefficients of the variables to progressively diminish near zero. In accordance with the standard of λ = 1, the optimal λ value was 0.0088, resulting in the selection of eight variables with non-zero coefficients: age, hyperlipidemia, atrial fibrillation and other cardiac diseases, pre-stroke statin use, antiplatelet/anticoagulation therapy within 48 h, admission ADL score, dysphagia, and D-dimer level. These variables were later incorporated into the model development.

### 3.3. Evaluation of the Modeling Cohort and Validation Cohort Model Performance

To assess the efficacy and generalizability of the developed models, the dataset was partitioned into a modeling cohort (for model creation and internal validation) and an independent validation cohort (for temporal validation). The modeling cohort was utilized to train all the ML models and evaluate their internal performance by 10-fold cross-validation, whereas the validation cohort was employed to examine model generalizability in an independent cohort.

#### 3.3.1. Evaluation of Modeling Cohort Model Performance

**Figure 4:**
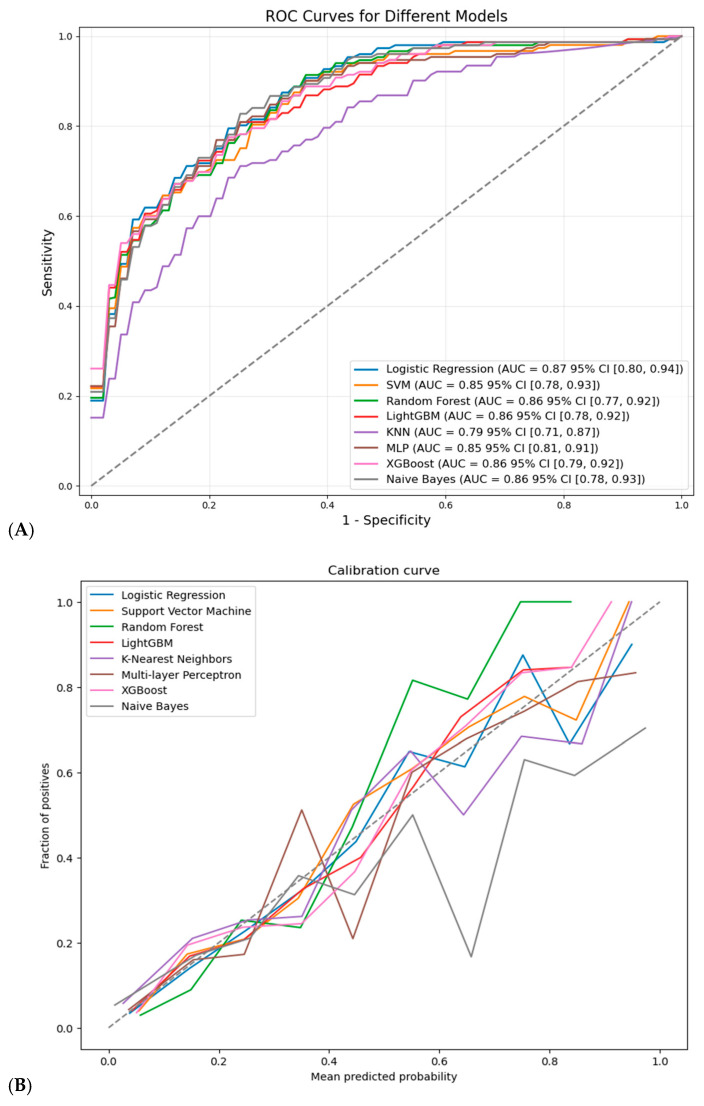

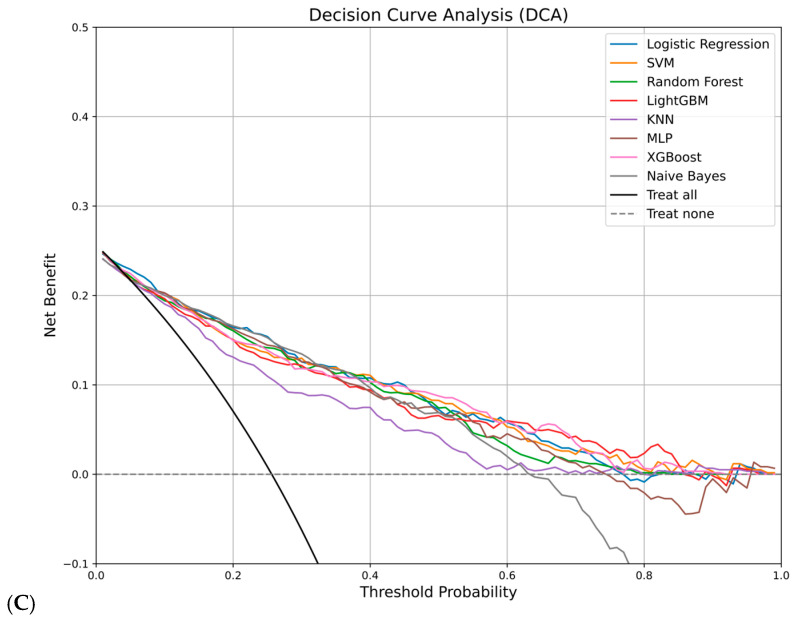
Modeling cohort model performance evaluation diagram: (**A**) ROC curves for individual models in the modeling cohort; (**B**) calibration curves for individual models in the modeling cohort; (**C**) DCA curves for individual models of the modeling cohort.

The data in Figure 4A demonstrate that all the models displayed robust discriminative ability, with the LR model attaining the maximum discriminatory power (AUC = 0.87; 95% CI: 0.80–0.94). The data in Table 3 indicate that the RF model exhibited the greatest sensitivity, whereas the MLP model attained the highest specificity, accuracy, and F1 score. The results of the model calibration are shown in Figure 4B. The calibration curve of XGBoost aligns most closely with the diagonal line; the SVM has a calibration slope nearest to 1, and the LR model has a calibration intercept closest to 1 and the lowest Brier score, indicating the most favorable overall calibration performance. The DCA depicted in Figure 4C indicated that the KNN and naive Bayes models yielded greater net benefits than the “treat-all” and “treat-none” strategies did within a threshold probability range of approximately 0.1–0.6, whereas the other models exhibited greater net benefits across a wider threshold probability range of approximately 0.1–0.8. Compared with the other ML models, the LR model had the greatest net benefit across most of the threshold probability ranges. For example, with a threshold probability of 0.3, the net benefit of the LR model was approximately 0.12, which was greater than that of the “treat-all” strategy, where the net benefit was negative and superior to that of the other ML models.

#### 3.3.2. Evaluation of Validation Group Model Performance

**Figure 5:**
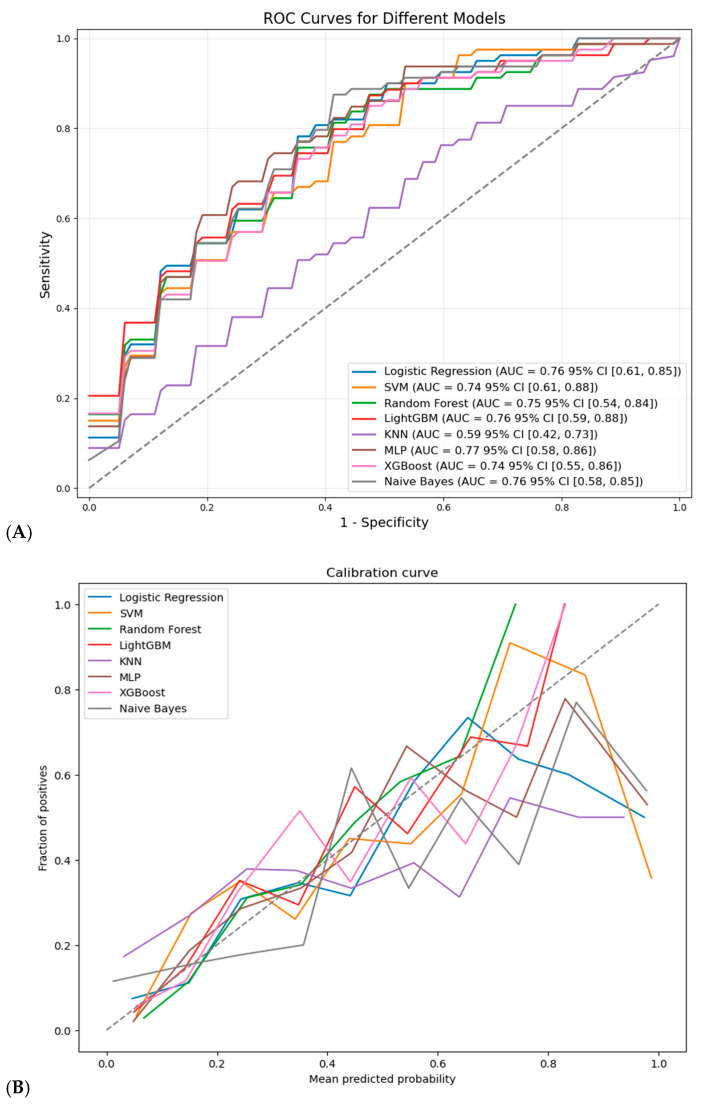

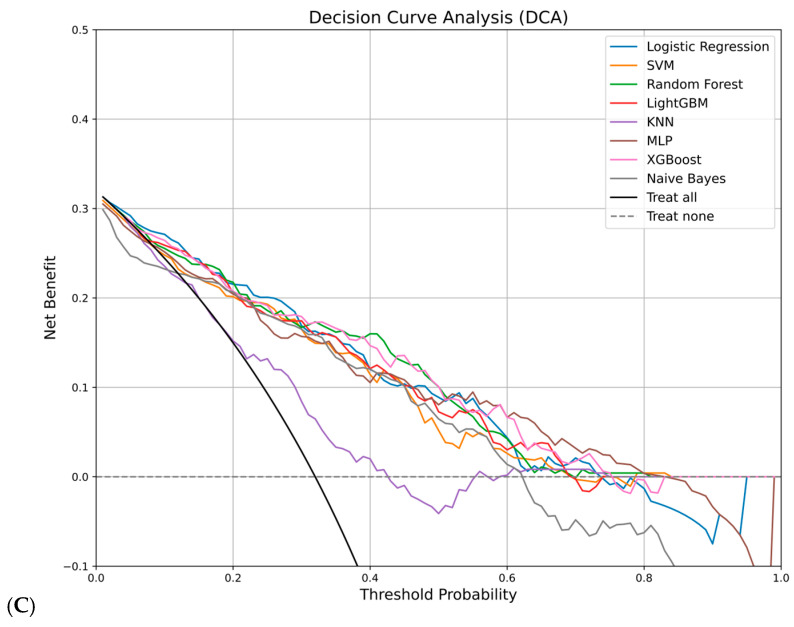
Validation cohort model performance evaluation diagram: (**A**) ROC curves for individual models in the validation cohort; (**B**) calibration curvesfor individual models in the validation cohort; (**C**) DCA curves for individual models of the validation cohort.

An overall drop in prediction performance was noted across all models when compared to the modelling group. Figure 5A illustrates that the ROC curves reveal the MLP model exhibited enhanced discriminative efficacy (AUC = 0.77, 95% CI: 0.58–0.86). In Table 4, the performance metrics indicate that the LightGBM model exhibited the maximum sensitivity, while the Naive Bayes model achieved the highest specificity, accuracy, and F1 score. Figure 5B demonstrates that the calibration curve of the LightGBM model is nearer to the diagonal. The XGBoost model attained the minimal Brier score, with its calibration slope and intercept nearest to 1. Figure 5C illustrates that the DCA revealed the KNN model yielded a superior net benefit compared to the “treat-all” and “treat-none” strategies within a threshold probability range of approximately 0.1–0.4, the MLP model within approximately 0.1–0.8, and the other models within approximately 0.1–0.7. The MLP model had the greatest net benefit across the majority of threshold probability ranges in comparison to the other ML models. At a threshold probability of 0.6, the net benefit of the MLP model was roughly 0.08, surpassing the negative net benefit of the “treat-all” method and outperforming other ML models.

### 3.4. Interpretation of the Model Analyses

Upon examining the performance metrics of each model in the modeling cohort and the temporal validation cohort, we determined that no individual model consistently surpassed the others across all the assessment metrics. Despite the LR model exhibiting robust discriminatory ability in the modeling cohort and several models displaying superiority in particular measures, its performance fluctuated across other datasets. The MLP model exhibited notably improved and more balanced prediction performance in both the modeling and temporal validation sets. We subsequently employed the SHAP technique to elucidate the MLP model.

**Figure 6:**
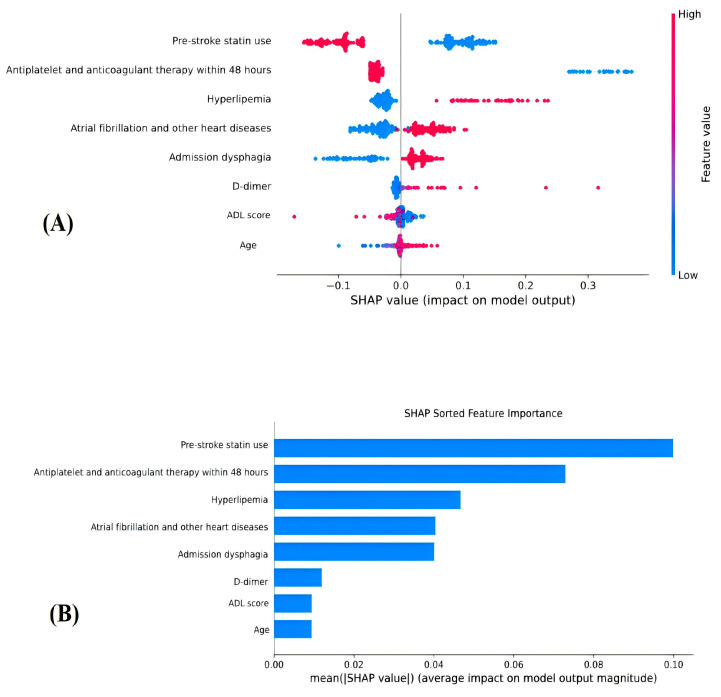
Distribution and ranking of feature SHAP values: (**A**) distribution of feature SHAP values; (**B**) distribution of feature importance. Note: (1) Positions on the vertical axis in Figure (**A**) are determined by the clinical features ranked in order of importance, and positions on the horizontal axis are determined by the SHAP values; (2) Each sample in Figure (**A**) is represented by a dot, the greater the dispersion of the dots is, the more pronounced the effect of the feature, and the color indicates the height of the feature value (high in red, low in blue); (3) Figure (**B**) shows a conventional bar chart in which each row corresponds to a characteristic, and a higher SHAP value indicates a higher risk of death.

The distribution and ranking of the SHAP values for the clinical characteristics are shown in Figure 6. The impact of these feature values on the outcomes of death predictions is shown in Figure 6A. For example, pre-stroke statin utilization is indicated by red dots, which primarily cluster in areas with negative SHAP values. This suggests that prior statin use is linked to a decreased probability of mortality post-stroke; in contrast, the lack of statin use (shown by blue dots) is associated with an elevated risk of death. A bar chart of feature importance, arranged by ascending mean absolute SHAP values, demonstrating the degree of impact each clinical feature has on the model’s predictions, is shown in Figure 6B. According to the feature importance ranking, the primary predictors of 90-day post-MT mortality risk were pre-stroke statin use, early antiplatelet/anticoagulant therapy (within 48 h), hyperlipidemia, atrial fibrillation (or other cardiac disorders), and admission dysphagia.

**Figure 7:**
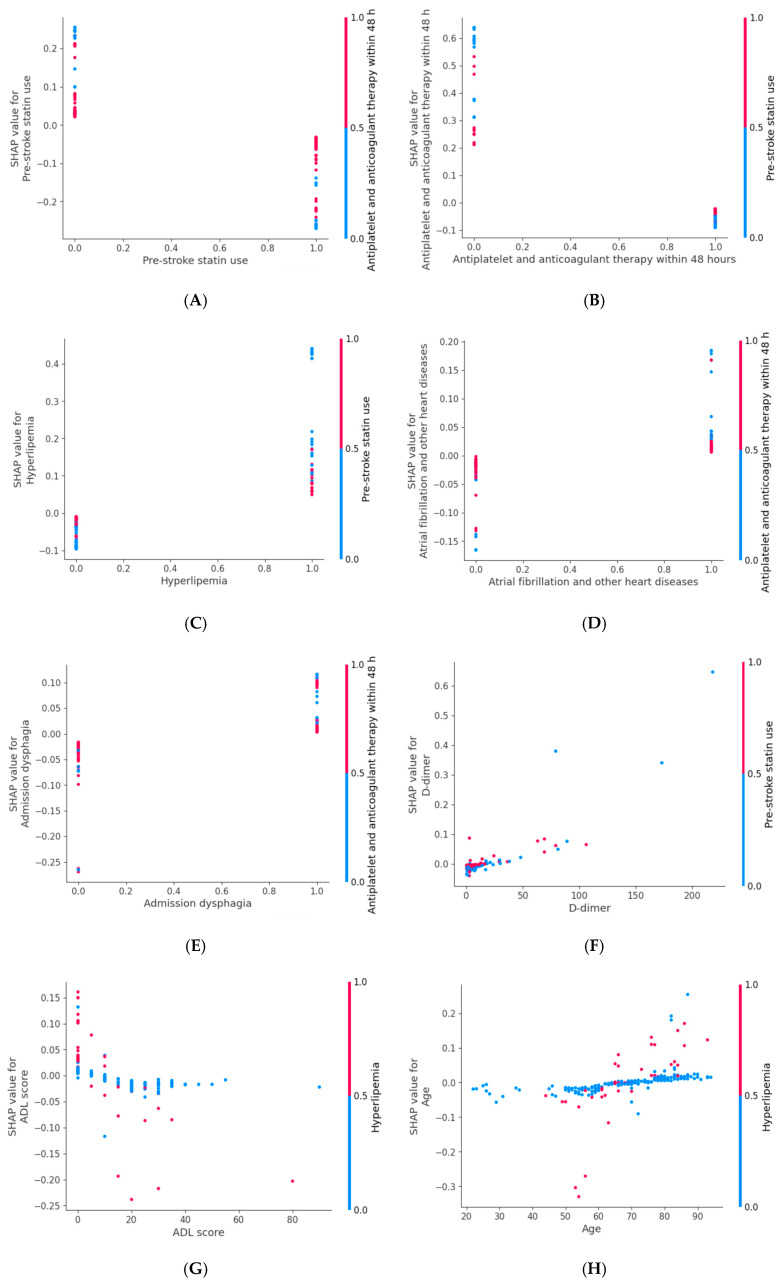
SHAP feature dependency graph. Note: In (**A**–**E**) the *x*-axis value of 0 means ‘no’ and 1 means ‘yes’, in (**F**–**H**) the *x*-axis is the actual value of the feature, and the *y*-axis on the left side is the value of the shape of each feature, and on the right side is the value of the second feature corresponding to the interaction of the feature being plotted.

The SHAP dependence plot (Figure 7) demonstrates the relationship between the features and the SHAP values of the model predictions, highlighting the influence of varying feature values on the predictions. The data in Figure 7C demonstrate that patients with hyperlipidemia display markedly elevated SHAP levels relative to those without the disease. This trait influences the administration of lipid-modifying medications, as patients utilizing these treatments exhibit a decreased risk of mortality. In Figure 7H, the influence of age on mortality risk in individuals with MT appears to be largely stable. Nonetheless, in association with the characteristics of hyperlipidemia, starting at age 50, advancing age is correlated with an increased SHAP value, hence heightening the mortality risk among these patients.

## 4. Discussion

In this study, eight independent predictors of 90-day mortality in patients who underwent MT were identified. We created and evaluated several ML models utilizing clinical data. Of the eight algorithms assessed, the MLP model exhibited superior overall performance in both internal and temporal validation, with relatively better discrimination, calibration, and clinical utility. Furthermore, model interpretability was augmented by the SHAP method, which allowed for the visualization of the contributions of individual predictors, thereby enhancing transparency and aiding clinical integration.

In this study, the mortality rates in the modeling and validation cohorts ranged from 25.6% to 32%, which is consistent with the findings of other studies [11,25]. Numerous studies have reported comparably elevated mortality rates, underscoring the necessity of investigating factors that affect mortality in MT patients and creating a comprehensive mortality risk prediction model. Significantly, differences were detected between the modeling and validation cohorts for the ADL score, serum albumin concentration and serum CRP concentration, monocyte count, and D-dimer level. These disparities may be ascribed to individual variability in the indicators, patients’ underlying illnesses, and therapeutic measures. Despite these variances, only two parameters (ADL score and D-dimer) that exhibited substantial differences were ultimately incorporated into the model. Nevertheless, the model’s performance remained consistent, and these disparities did not significantly impact its predictive capability. These findings suggest that the model retained relatively stable predictive performance despite certain inter-cohort differences.

Through univariate and LASSO regression models, this study revealed multiple predictors of death in patients with AIS who received MT. Among these parameters, age proved to be a major prognostic predictor, with advancing age worsening the mortality risk in AIS patients [26]. Older patients typically face a heightened risk of comorbidities and demonstrate a reduced ability for cerebrovascular repair, both of which increase mortality risk [27]. Previous research has shown that cardiovascular disorders, including hyperlipidemia and atrial fibrillation, increase the severity of AIS and increase death rates, supporting the conclusions of this study [28,29]. Hyperlipidemia and other cardiac disorders, such as atrial fibrillation, not only increase the risk of AIS but also aggravate the pathophysiological mechanisms following MT by compromising vascular endothelial function and disrupting hemodynamics.

Research conducted by M’barek et al. [30] demonstrated that elevated D-dimer levels upon admission increased the 1-year mortality risk in patients with AIS by 1.55 times, corroborating the findings of the current study. Elevated D-dimer, a biomarker reflecting coagulation and fibrinolytic activity, is correlated with an increased risk of thrombotic events and stroke recurrence. Dysphagia at admission is a prevalent complication among stroke patients. This study revealed a significant association between dysphagia and mortality risk in patients who underwent MT, supporting the conclusions of Li et al. [31] Thus, the prompt recognition and treatment of dysphagia may improve patient outcomes.

This study demonstrated that pre-stroke statin utilization is correlated with a reduced mortality risk. Prior research has indicated that pre-stroke statin administration may increase anti-inflammatory, antioxidant, and atherosclerotic plaque stabilization and improve vascular function, thus alleviating neurological symptoms and decreasing mortality risk [32,33]. The current investigation revealed that antiplatelet and anticoagulant medication administered within 48 h was linked to a decreased mortality risk in patients with MT. Numerous extensive clinical trials, including TIMING [34] and ELAN [35], have substantiated the significance of early antiplatelet and anticoagulant therapy. Nonetheless, the existing evidence remains inadequate to definitively endorse the vigorous pursuit of early anticoagulation in patients with MT. Consequently, clinicians must select a suitable therapeutic regimen on the basis of the patient’s particular condition. This study revealed that a higher ADL score at admission was inversely correlated with the likelihood of mortality. The ADL score serves as a measure of functional state, indicating overall illness condition, neurological impairments, and rehabilitation potential, thereby holding clinical significance for determining mortality risk [36].

Despite the NIHSS score being a recognized measure of stroke severity and its extensive correlation with prognosis in AIS patients, it was excluded from the final model following LASSO regression. An explanation may be that the predicted information provided by the NIHSS score coincided with other retained characteristics associated with neurological impairment and functional status, notably the entry ADL score and dysphagia. LASSO regression selects variables that provide the greatest incremental predictive value while reducing redundancy and model complexity. Consequently, clinically significant variables may not persist in the final model following penalization. The resulting model exhibited adequate discrimination and calibration performance in both the internal and external validation datasets, suggesting that the omission of the NIHSS did not substantially impair model performance within the current dataset.

This study involved the construction and evaluation of eight ML models to assess their efficacy in predicting the prognosis of stroke patients. In comparison to the other models, the MLP models demonstrated relatively better predictive performance in both the modeling cohort and the temporal validation set, achieving AUC values of 0.85 and 0.77, respectively, thereby underscoring the efficacy of the MLP models in capturing intricate nonlinear relationships and feature interactions [37]. A decline in model performance between the modeling and validation cohorts was noted, a phenomenon that is relatively common in clinical prediction modeling [21] and may be linked to variations in data distribution between cohorts, multicenter heterogeneity, and the inherent complexity of ML algorithms [38]. Furthermore, the implementation of 10-fold cross-validation during model development may have helped reduce overfitting and improve the stability of the performance estimation. Nevertheless, because neither nested cross-validation nor bootstrap optimism correction was performed, some degree of model optimism cannot be excluded. Although the models maintained acceptable predictive performance in the temporal validation cohort, further multicenter external validation is still needed to confirm their generalizability.

The predictive models developed by Li [39] and Matsukawa et al. [18] utilizing the LR algorithm for predicting mechanical thrombus extraction outcomes achieved AUCs of 0.87 and 0.79, respectively, during internal validation, demonstrating strong performance and significant clinical value. Tong et al. [40] effectively employed the XGBoost algorithm to predict the prognosis of MT patients, achieving AUCs of 0.93 and 0.77 for the model test set and validation set, respectively. Compared with previous studies, the MLP model in this research demonstrated comparable predictive performance. This performance was further confirmed by temporal validation, highlighting its potential utility for clinical decision-making and prognostic evaluation in stroke patients. Notably, in the modeling cohort, the LR model exhibited robust discrimination and satisfactory calibration; nevertheless, its net therapeutic benefit was comparatively diminished in the DCA. This mismatch can be attributed to the fundamental assumption of linear relationships in linear regression models, which constrains their capacity to include intricate nonlinear effects and interactions among predictors [41]. Conversely, more adaptable ML models, such as MLPs, can more effectively capture intricate patterns, resulting in enhanced clinical decision-making ability across a broader spectrum of threshold probabilities [42].

In contrast to conventional ML models grounded in linear regression, such as LR, some advanced ML models—such as the MLP employed in this study—are regarded as “black-box” models owing to their intricate internal architectures and opacity. This paper presents the SHAP algorithm to improve interpretability and ensure transparency in healthcare applications. This novel approach for evaluating diverse black-box ML models offers both local and global insights, clarifies the impact of specific variables on the model’s predictive capability, and fosters trust between physicians and artificial intelligence algorithms [43,44]. An interpretative analysis of the MLP model utilizing the SHAP algorithm indicated that pre-stroke statin use, antiplatelet/anticoagulant therapy within 48 h, hyperlipidemia, the presence of atrial fibrillation and other cardiac diseases, and admission dysphagia were significant factors enhancing the model’s predictive efficacy. Various findings indicate the proportional impact of various variables on the model’s decision-making process. This information can be used to facilitate clinicians’ understanding of individual risk forecasts and to promote better informed and individualized clinical decision-making. It is important to note that SHAP analysis is a model-agnostic interpretability tool that elucidates the contribution of each variable to the model’s predictive output rather than determining causal links between variables and clinical outcomes.

This study offers four primary contributions. First, rather than relying on a single algorithm, we developed and evaluated eight conventional ML models to predict 90-day mortality following MT. Second, we integrated 10-fold cross-validation with temporal validation to rigorously assess model stability and generalizability, thereby providing a more comprehensive assessment of model performance and potential clinical utility. Third, the models were comprehensively evaluated on the basis of discrimination, calibration, and clinical utility. Fourth, the SHAP method was employed to elucidate the primary predictors, thereby facilitating personalized clinical decision-making. Collectively, this clinically oriented framework—encompassing model comparison, multistage validation, and interpretability analysis—enhances the translational value of ML in prognostic prediction for stroke patients.

This study has several limitations. First, its single-center design and the use of a temporal validation cohort from the same institution may introduce selection bias. Furthermore, the reliance on a population primarily from one region in China potentially limits the generalizability and fairness of the model across diverse ethnic cohorts, hospital settings, and healthcare systems. Consequently, external validation using multicenter and multiethnic cohorts is warranted to rigorously evaluate the robustness of the model. In addition, although temporal validation was performed, both the modeling and validation cohorts originated from the same institution. Therefore, the current validation strategy cannot fully eliminate the possibility of model optimism or institutional bias. Second, despite the inclusion of numerous clinical variables, certain critical imaging predictors typically utilized in thrombectomy prognosis studies were omitted because of data inconsistency inherent in this retrospective design. Similarly, the lack of granular data concerning operator experience may leave residual procedural confounding. Future multicenter investigations incorporating standardized imaging parameters and detailed procedural metrics are essential for enhancing model performance. Third, while univariate pre-selection prior to LASSO regression may have excluded predictors with weak univariate but significant multivariable associations, this step served as an initial dimensionality reduction technique, followed by rigorous refinement via LASSO and ML. Future studies with larger datasets could explore alternative variable-selection strategies to validate our approach. Fourth, pre-stroke statin and antiplatelet/anticoagulant use may introduce confounding effects, as these factors likely reflect baseline health status, healthcare access, or stroke characteristics. Furthermore, the reliance on binary (yes/no) data—lacking information on dosage, duration, or adherence—may result in exposure misclassification. Finally, residual confounding remains a concern. Owing to the retrospective nature of this study, data concerning ICU and in-hospital complications (e.g., pneumonia, aspiration, infections, mechanical ventilation duration, and cardiac events) were not consistently available and thus excluded, potentially affecting the comprehensiveness of the model.

## 5. Conclusions

In this study, we developed and validated a prognostic model for 90-day mortality in patients who underwent MT. Eight independent predictors were identified: age, hyperlipidemia, atrial fibrillation (or other cardiac disorders), pre-stroke statin use, antiplatelet/anticoagulant therapy within 48 h, admission dysphagia, ADL score, and D-dimer level. Among the evaluated algorithms, the MLP model demonstrated relatively favorable predictive performance within the current dataset in terms of discrimination, calibration, and clinical utility. These findings may support individualized risk assessment for patients undergoing MT. However, given the single-center retrospective design and the absence of independent multicenter external validation, the model should be interpreted cautiously prior to clinical implementation.

## Figures and Tables

**Table 1:** Baseline clinical characteristics of patients in the modeling cohort [M * (IQR, 25, 75), *n* (%)].

Variables	Survivor Group (*n* = 441)	Death Group (*n* = 152)	*U* ^†^/*x*^2 ‡^/Fisher ^§^	*p*
Age	70 (58, 78)	76.5 (67.25, 82)	5.356	<0.001
Gender			4.185	0.041
Female	187 (42.4%)	79 (52%)		
Male	254 (57.6%)	73 (48%)		
Ethnicity			Fisher	0.963
Han Chinese	433 (98.2%)	150 (98.7%)		
Other minorities	8 (1.8)	2 (1.3%)		
BMI (kg/m^2^)	23.44 (21.48, 25.66)	22.99 (20.55, 25.39)	−1.787	0.074
Educational background			12.408	0.053
Illiteracy	31 (7.0%)	21 (13.8%)		
Primary school	134 (30.4%)	56 (36.8%)		
Junior middle school	111 (25.2%)	34 (22.4%)		
High school	48 (10.9%)	15 (9.9%)		
Vocational secondary school	33 (7.5%)	9 (5.9%)		
Junior college	32 (7.2%)	6 (3.9%)		
Undergraduate or above	52 (11.8%)	11 (7.3%)		
Smoking history			0.232	0.63
No	316 (71.7%)	112 (73.7%)		
Yes	125 (28.3%)	40 (26.3%)		
Drinking history			0.033	0.855
No	311 (70.5%)	106 (69.7%)		
Yes	130 (29.5%)	46 (30.3%)		
History of stroke			2.119	0.146
No	406 (92.1%)	134 (88.2%)		
Yes	35 (7.9%)	18 (11.8%)		
TIA			Fisher	0.972
No	436 (98.9%)	151 (99.3%)		
Yes	5 (1.1%)	1 (0.7%)		
Hypertension			0.087	0.768
No	171 (38.8%)	61 (40.1%)		
Yes	270 (61.2%)	91 (59.9%)		
Diabetes			0.484	0.487
No	336 (76.2%)	120 (78.9%)		
Yes	105 (23.8%)	32 (21.1%)		
Hyperlipemia			17.069	<0.001
No	379 (85.9%)	108 (71.1%)		
Yes	62 (14.1%)	44 (28.9%)		
Atrial fibrillation and other cardiac diseases			73.435	<0.001
No	298 (67.6%)	58 (38.2%)		
Yes	143 (32.4%)	94 (61.8%)		
Atherosclerosis			3.722	0.054
No	360 (81.6%)	113 (74.3%)		
Yes	81 (18.4%)	39 (25.7%)		
Temperature	36.5 (36.3, 36.6)	36.5 (36.3, 36.6)	0.183	0.855
Pulse	78 (68, 87)	79 (72, 90)	1.767	0.077
Respiration	19 (14, 20)	18 (14, 20)	−1.741	0.082
Systolic pressure	134 (118, 150)	134 (117, 153)	0.216	0.829
Diastolic pressure	76 (68, 87)	76 (66, 87)	−1.127	0.26
TOAST typology			2.175	0.717
Atherosclerotic sclerotic type	240 (54.4%)	79 (52%)		
Cardioembolic type	131 (29.7%)	53 (34.8%)		
Arteriole occlusion type	5 (1.1%)	1 (0.7%)		
Other etiology-identified type	18 (4.1%)	7 (4.6%)		
Unknown cause type	47 (10.7%)	12 (7.9%)		
Vascular occlusion site			2.000	0.368
ICA	137 (31.1%)	49 (32.2%)		
MCA	228 (51.7%)	70 (46.1%)		
BA + VA	76 (17.2%)	33 (21.7%)		
NIHSS score			25.93	<0.001
Mild < 4 points	20 (4.5%)	1 (0.7%)		
Moderate < 16 points	266 (60.3%)	68 (44.7%)		
Severe < 25 points	121 (27.5%)	54 (35.5%)		
Very severe ≥ 25 points	34 (7.7%)	29 (19.1%)		
mRS score			6.466	0.249
Level 0	10 (2.3%)	0 (0%)		
Level 1	5 (1.1%)	3 (2%)		
Level 2	15 (3.4%)	4 (2.6%)		
Level 3	17 (3.9%)	4 (2.6%)		
Level 4	114 (25.8%)	33 (21.7%)		
Level 5	280 (63.5%)	108 (71.1%)		
ADL score	20 (0, 50)	0 (0, 10)	−9.188	<0.001
Admission dysphagia			76.893	<0.001
No	211 (47.8%)	12 (7.9%)		
Yes	230 (52.2%)	140 (92.1%)		
Pre-stroke statin use			28.361	<0.001
No	117 (26.5%)	76 (50%)		
Yes	324 (73.5%)	76 (50%)		
Pre-stroke anticoagulants use			15.157	<0.001
No	275 (62.4%)	121 (79.6%)		
Yes	166 (37.6%)	31 (20.4%)		
Pre-stroke antiplatelet use			28.939	<0.001
No	86 (19.5%)	63 (41.4%)		
Yes	355 (80.5%)	89 (58.6%)		
Antiplatelet/anticoagulant therapy within 48 h			47.73	<0.001
No	63 (14.3%)	62 (40.8%)		
Yes	378 (85.7%)	90 (59.2%)		
Bridging treatment			1.274	0.259
No	369 (83.7%)	133 (87.5%)		
Yes	72 (16.3%)	19 (12.5%)		
Methods of thrombectomy			6.016	0.049
Aspiration thrombectomy	186 (42.2%)	47 (30.9%)		
Stent retriever thrombectomy	132 (29.9%)	55 (36.2%)		
Aspiration thrombectomy + Stent retriever thrombectomy	123 (27.9%)	50 (32.9%)		
Number of thrombus pulls			15.954	<0.001
1 time	242 (54.9%)	58 (38.2%)		
2–3 times	154 (34.9%)	64 (42.1%)		
>3 times	45 (10.2%)	30 (19.7%)		
Anesthesia methods			0.428	0.513
Local anesthetic	83 (18.8%)	25 (16.4%)		
General anesthetic	358 (81.2%)	127 (83.6%)		
Anesthesia grading			23.242	<0.001
Level 2	21 (4.8%)	3 (2%)		
Level 3	361 (81.8%)	103 (67.7%)		
Level 4	59 (13.4%)	46 (30.3%)		
OPT	310 (240, 471)	306.5 (234.3, 430)	−0.66	0.509
PNT	60 (40, 90)	71 (44, 108)	2.528	0.011
Endotracheal Intubation			1.581	0.209
No	87 (19.7%)	23 (15.1%)		
Yes	354 (80.3%)	129 (84.9%)		
Balloon dilation/stenting			4.571	0.033
No	328 (74.4%)	126 (82.9%)		
Yes	113 (25.6%)	26 (17.1%)		
Albumin	40.2 (37.8, 42.7)	39.2 (36.7, 42)	−2.341	0.019
Lymphocyte count (10^9^/L)	1.1 (0.83, 1.6)	0.88 (0.6, 1.43)	−3.871	<0.001
CRP (mg/L)	10.99 (8.11, 22.15)	18.53 (8.18, 44.26)	3.339	<0.001
Total cholesterol (mmol/L)	4.08 (3.4, 4.78)	4.14 (3.51, 4.76)	0.579	0.563
Monocyte count (10^9^/L)	0.53 (0.39, 0.71)	0.54 (0.37, 0.78)	0.438	0.661
Neutrophil count (10^9^/L)	6.89 (4.76, 9.45)	7.31 (4.86, 10.96)	1.789	0.074
Platelet count (10^9^/L)	169 (136, 211)	163 (126, 214)	−0.885	0.376
Uric acid (umol/L)	309 (242, 381)	337 (238, 401)	1.435	0.151
D-dimer (mg/L FEU)	1.38 (0.62, 2.44)	2.54 (1.37, 8.45)	6.667	<0.001
Fibrinogen (g/L)	2.76 (2.29, 3.25)	2.85 (2.28, 3.59)	0.964	0.335
Triglycerides (mmol/L)	1.16 (0.85, 1.76)	1.13 (0.82, 1.56)	−0.537	0.591
Low-density lipoprotein cholesterol (mmol/L)	2.4 (1.79, 3)	2.36 (1.95, 2.97)	0.261	0.794
High-density lipoprotein cholesterol (mmol/L)	1.19 (0.98, 1.44)	1.25 (1.06, 1.48)	1.471	0.141

*: M, median. ^†^: U, Mann-Whitney U test. ^‡^: x^2^, chi-square test. ^§^: Fisher, Fisher’s exact test. Abbreviations: (1) IQR, interquartile range; (2) BMI, body mass index; (3) TIA, transient ischemic attack; (4) ICA, internal carotid artery; (5) MCA, middle cerebral artery; (6) BA, basilar artery; (7) VA, vertebral artery.

**Table 2:** Comparison of baseline information between the modeling and validation cohorts [M * (IQR 25, 75), *n* (%)].

Variables	Modeling Cohort (*n* = 593)	Validation Cohort (*n* = 247)	*U* ^†^/*x*^2 ‡^	*p*
Age	71 (59.5, 79)	69 (59, 79)	−0.486	0.627
Gender			2.003	0.157
Female	266 (44.9%)	124 (50.2%)		
Male	327 (55.1%)	123 (49.8%)		
Ethnicity			1.234	0.267
Han Chinese	583 (98.3%)	236 (95.5%)		
Other minorities	10 (1.7%)	7 (2.8%)		
BMI (kg/m^2^)	23.44 (21.39, 25.59)	23.81 (21.97, 25.69)	1.188	0.235
Educational background			7.429	0.283
Illiteracy	52 (8.8%)	29 (11.7%)		
Primary school	190 (32.0%)	69 (27.9%)		
Junior middle school	145 (24.5%)	62 (25.2%)		
High school	63 (10.6%)	27 (10.9%)		
Vocational secondary school	42 (7.1%)	16 (6.5%)		
Junior college	38 (6.4%)	25 (10.1%)		
Undergraduate or above	63 (10.6%)	19 (7.7%)		
Smoking history			0.254	0.614
No	428 (72.2%)	180 (72.9%)		
Yes	165 (27.8%)	67 (27.1%)		
Drinking history			0.391	0.532
No	417 (70.3%)	179 (72.5%)		
Yes	176 (29.7%)	68 (27.5%)		
History of stroke			0.804	0.370
No	540 (91.1%)	220 (89.1%)		
Yes	53 (8.9%)	27 (10.9%)		
TIA			0.547	0.459
No	587 (99%)	243 (98.4%)		
Yes	6 (1%)	4 (1.6%)		
Hypertension			0.135	0.713
No	232 (39.1%)	100 (40.5%)		
Yes	361 (60.9%)	147 (59.5%)		
Diabetes			0.246	0.620
No	456 (76.9%)	186 (75.3%)		
Yes	137 (23.1%)	61 (24.7%)		
Hyperlipemia			0.197	0.657
No	487 (82.1%)	206 (83.4%)		
Yes	106 (17.9%)	41 (16.6%)		
Atrial fibrillation and other cardiac diseases			2.399	0.121
No	356 (60.0%)	134 (54.3%)		
Yes	237 (40.0%)	113 (45.7%)		
Atherosclerosis			0.846	0.358
No	473 (79.8%)	190 (76.9%)		
Yes	120 (20.2%)	57 (23.1%)		
Temperature	37 (36, 37)	37 (36, 37)	0.360	0.719
Pulse	78 (69, 88)	78 (70, 89)	1.002	0.317
Respiration	19 (14, 20)	19 (14, 20)	−0.168	0.867
Systolic pressure	134 (118, 151)	132 (117, 150)	−0.011	0.991
Diastolic pressure	76 (67, 87)	76 (67, 87)	−0.296	0.768
TOAST typology			1.045	0.903
Atherosclerotic sclerotic type	319 (53.8%)	140 (56.7%)		
Cardioembolic type	184 (31%)	74 (30%)		
Arteriole occlusion type	6 (1.1%)	3 (1.2%)		
Other etiology-identified type	25 (4.2%)	10 (4%)		
Unknown cause type	59 (9.9%)	20 (8.1%)		
Vascular occlusion site			0.744	0.689
ICA	186 (31.4%)	85 (34.4%)		
MCA	298 (50.3%)	119 (48.2%)		
BA + VA	109 (18.3%)	43 (17.4%)		
NIHSS score			4.702	0.195
Mild < 4 points	21 (3.6%)	16 (6.4%)		
Moderate < 16 points	334 (56.3%)	135 (54.7%)		
Severe < 25 points	175 (29.5%)	65 (26.3%)		
Very severe ≥ 25 points	63 (10.6%)	31 (12.6%)		
MRS score			8.694	0.122
Level 0	10 (1.7%)	6 (2.4%)		
Level 1	8 (1.3%)	6 (2.4%)		
Level 2	19 (3.2%)	11 (4.5%)		
Level 3	21 (3.6%)	17 (6.9%)		
Level 4	147 (24.8%)	65 (26.3%)		
Level 5	388 (65.4%)	142 (57.5%)		
ADL score	10 (0, 35)	0 (0, 20)	−5.916	<0.001
Admission dysphagia			3.555	0.059
No	223 (37.6%)	76 (30.8%)		
Yes	370 (62.4%)	171 (69.2%)		
Pre-stroke statin use			0.563	0.453
No	193 (32.5%)	87 (35.2%)		
Yes	400 (67.5%)	160 (64.8%)		
Pre-stroke anticoagulants use			0.849	0.357
No	396 (66.8%)	173 (70%)		
Yes	197 (33.2%)	74 (30%)		
Pre-stroke antiplatelet use			0.569	0.451
No	149 (25.1%)	56 (22.7%)		
Yes	444 (74.9%)	191 (77.3%)		
Antiplatelet/anticoagulant therapy within 48 h			0.020	0.889
No	125 (21.1%)	51 (20.6%)		
Yes	468 (78.9%)	196 (79.4%)		
Bridging treatment			0.546	0.460
No	502 (84.7%)	214 (86.6%)		
Yes	91 (15.3%)	33 (13.4%)		
Methods of thrombectomy			0.604	0.739
Aspiration thrombectomy	233 (39.3%)	90 (36.4%)		
Stent retriever thrombectomy	187 (31.5%)	82 (33.2%)		
Aspiration thrombectomy + Stent retriever thrombectomy	173 (29.2%)	75 (30.4%)		
Number of thrombus pulls			0.604	0.739
1 time	300 (50.6%)	135 (54.6%)		
2–3 times	218 (36.8%)	100 (40.5%)		
>3 times	75 (12.6%)	12 (4.9%)		
Anesthesia methods			1.185	0.276
Local anesthetic	108 (18.2%)	53 (21.5%)		
General anesthetic	485 (81.8%)	194 (78.5%)		
Anesthesia grading			3.364	0.186
Level 2	24 (4.1%)	14 (5.7%)		
Level 3	464 (78.2%)	179 (72.5%)		
Level 4	105 (17.7%)	54 (21.8%)		
OPT	310 (240, 452)	294 (230, 480)	−0.663	0.507
PNT	64 (40, 95)	67 (43, 100)	0.757	0.449
Endotracheal Intubation			2.244	0.134
No	110 (18.5%)	57 (23.1%)		
Yes	483 (81.5%)	190 (76.9%)		
Balloon dilation/stenting			3.737	0.053
No	454 (76.6%)	204 (82.6%)		
Yes	139 (23.4%)	43 (17.4%)		
Albumin	40.1 (37.4, 42.6)	39.1 (36.3, 41.9)	−3.178	0.001
Lymphocyte count (10^9^/L)	1.06 (0.79, 1.54)	1.06 (0.69, 1.44)	−1.443	0.149
CRP (mg/L)	11.6 (8.11, 28.45)	10.13 (6.16, 21.9)	−2.652	0.008
Total cholesterol (mmol/L)	4.08 (3.46, 4.78)	3.96 (3.38, 4.7)	−1.098	0.272
Monocyte count (10^9^/L)	0.53 (0.38, 0.72)	0.58 (0.4, 0.76)	2.111	0.035
Neutrophil count (10^9^/L)	7.01 (4.77, 9.62)	7.57 (5.66, 10.45)	2.534	0.011
Platelet count (10^9^/L)	168 (134, 211.5)	169 (126, 216)	−0.137	0.891
Uric acid (umol/L)	313 (242, 384)	302 (235, 382)	−1.025	0.305
D-dimer (mg/L FEU)	1.38 (0.71, 3.4)	1.71 (0.92, 4.48)	3.009	0.003
Fibrinogen (g/L)	2.79 (2.29, 3.32)	2.73 (2.3, 3.33)	−0.490	0.624
Triglycerides (mmol/L)	1.15 (0.85, 1.71)	1.15 (0.82, 1.64)	−0.454	0.650
Low-density lipoprotein cholesterol (mmol/L)	2.39 (1.83, 2.99)	2.4 (1.8, 2.94)	−0.266	0.790
High-density lipoprotein cholesterol (mmol/L)	1.2 (1, 1.46)	1.2 (1.01, 1.47)	−0.153	0.878

*: M, median. ^†^: U, Mann-Whitney U test. ^‡^: x^2^, chi-square test. Abbreviations: (1) IQR, interquartile range; (2) BMI, body mass index; (3) TIA, transient ischemic attack; (4) ICA, internal carotid artery; (5) MCA, middle cerebral artery; (6) BA, basilar artery; (7) VA, vertebral artery.

**Table 3:** Individual model evaluation indicators for the modeling cohort.

Models	Optimal Threshold	AUC	Sensitivity	Specificity	Accuracy	F1 Score	Calibration Slope	Calibration Intercept	Brier Score
LR	0.250	0.87	0.836	0.764	0.783	0.663	0.959	0.011	0.124
SVM	0.289	0.85	0.737	0.816	0.796	0.649	0.978	0.019	0.128
RF	0.252	0.86	0.842	0.717	0.749	0.632	1.345	−0.090	0.130
LightGBM	0.269	0.86	0.776	0.773	0.774	0.638	1.088	−0.029	0.128
KNN	0.236	0.79	0.763	0.678	0.699	0.566	0.877	0.044	0.152
MLP	0.328	0.85	0.750	0.828	0.808	0.667	0.914	0.015	0.125
XGBoost	0.238	0.86	0.796	0.751	0.762	0.632	1.105	−0.038	0.127
Naive Bayes	0.250	0.86	0.841	0.746	0.771	0.653	0.618	0.061	0.147

**Table 4:** Individual model evaluation indicators for the validation cohort.

Models	Optimal Threshold	AUC	Sensitivity	Specificity	Accuracy	F1 Score	Calibration Slope	Calibration Intercept	Brier Score
LR	0.222	0.76	0.899	0.542	0.656	0.626	0.549	0.156	0.155
SVM	0.249	0.74	0.772	0.625	0.672	0.601	1.171	−0.031	0.167
RF	0.256	0.76	0.873	0.530	0.640	0.608	1.298	−0.084	0.152
LightGBM	0.188	0.76	0.911	0.500	0.632	0.613	1.128	−0.063	0.159
KNN	0.188	0.60	0.798	0.417	0.539	0.525	0.867	0.097	0.210
MLP	0.283	0.77	0.798	0.649	0.696	0.627	0.871	0.047	0.153
XGBoost	0.294	0.74	0.760	0.673	0.700	0.619	1.079	−0.022	0.151
Naive Bayes	0.397	0.76	0.785	0.696	0.725	0.646	0.582	0.175	0.184

## Data Availability

Due to privacy restrictions, all raw data and code generated or analysed during this study are available from the corresponding author upon reasonable request.

## Abbreviations

The following abbreviations are used in this manuscript:

AIS	Acute Ischemic Stroke
MT	Mechanical Thrombectomy
ML	Machine Learning
SHAP	Shapley Additive Explanations
mTICI	modified Thrombolysis in Cerebral Infarction
AUC	area under the curve
NIHSS	National Institutes of Health Stroke Scale
mRS	modified Rankin Scale
ADL	activities of daily living
LR	logistic regression
SVM	Support Vector Machine
RF	Random Forest
LightGBM	Light Gradient Boosting Machine
KNN	K-Nearest Neighbors
MLP	Multi-Layer Perceptron
XGBoost	eXtreme Gradient Boosting
ROC	receiver operating characteristic
DCA	decision curve analysis
